# Effect of Immunomodulating Extract and Some Isolates from *Etlingera rubroloba* A.D. Poulsen Fruits on Diabetic Patients with Tuberculosis

**DOI:** 10.3390/molecules28052401

**Published:** 2023-03-06

**Authors:** Muhammad Ilyas Y., Idin Sahidin, Asriullah Jabbar, Agung W. M. Yodha, Ajeng Diantini, Ivan Surya Pradipta, Riezki Amalia, Raden Maya Febrianti, Yuni Elsa Hadisaputri, Mohammad Ghozali, Euis Julaeha

**Affiliations:** 1 Doctoral Program in Pharmacy, Faculty of Pharmacy, Universitas Padjadjaran, Jl. Raya Bandung-Sumedang KM 21, Sumedang 45363, Indonesia; 2 Faculty of Pharmacy, Universitas Halu Oleo, Kendari 93232, Indonesia; 3 Politeknik Bina Husada Kendari, Jl. Sorumba No.17, Kendari 93117, Indonesia; 4 Department of Pharmacology and Clinical Pharmacy, Faculty of Pharmacy, Universitas Padjadjaran, Jl. Raya Bandung-Sumedang KM 21, Sumedang 45363, Indonesia; 5 Department of Biomedical Sciences, Faculty of Medicine, Universitas Padjadjaran, Jl. Raya Bandung-Sumedang KM 21, Sumedang 45363, Indonesia; 6 Faculty of Mathematics and Natural Sciences, University of Padjadjaran, Jl. Raya Bandung-Sumedang KM 21, Sumedang 45363, Indonesia

**Keywords:** *Etlingera rubroloba* fruit isolate, Interleukin-12, Toll-like receptor-2, human leucocyte antigen-DR, macrophages, immunomodulator, diabetes mellitus, tuberculosis antigen

## Abstract

Diabetes mellitus (DM) is a disease easily complicated by tuberculosis (TB) due to impaired function of the innate immune response. The successes of the discovery of immunomodulatory compounds needs to be continued to introduce new insights into the innate immune response. In previous studies, plant compounds of *Etlingera rubroloba* A.D. Poulsen (*E.rubroloba*) were demonstrated to have potential as an immunomodulators. This study aims to isolate and identify the structure of the compounds of *E.rubroloba* fruit that could effectively improve the function of the innate immune response in individuals with DM infected with TB. The isolation and purification of the compounds of the *E.rubroloba* extract were carried out by radial chromatography (RC) and thin-layer chromatography (TLC). Identification of the isolated compound structures was determined by measuring the proton (^1^H) and carbon (^13^C) nuclear magnetic resonance (NMR). In vitro testing was performed on the immunomodulating activity of the extracts and isolated compounds on DM model macrophages infected with TB antigens. This study succeeded at isolating and identifying the structures of two isolate compounds, namely Sinaphyl alcohol diacetat (BER-1), and Ergosterol peroxide (BER-6). The two isolates were more effective as immunomodulators than the positive controls were, which differed significantly (* *p* < 0.05) at the reducing interleukin-12 (IL-12) levels and Toll-like receptor-2 (TLR-2) protein expression and increasing the human leucocyte antigen-DR (HLA-DR) protein expression in DM infected with TB. The isolated compound was discovered in *E. rubroloba* fruits, which has been reported to have the potential to be developed as an immunomodulatory agent. Follow-up testing to determine the mechanism and effectiveness of these compounds as immunomodulators for DM patients is required so that they are not susceptible to TB infection.

## 1. Introduction

Diabetes mellitus (DM) is a major health problem that has received great attention around the world, as it causes 70% of the total deaths [1]. The global DM epidemic causes increasing economic losses, especially in developing countries, such as countries in Asia and Africa. The 2017 International Diabetes Federation (IDF) report shows that global DM treatment costs total more than USD 727 billion per year or around 12 percent of global health financing [2], and there has been a significant increase in the DM handling costs.

A complication of DM that often arises from severe infectious diseases is tuberculosis (TB), which contributes to increasing morbidity and mortality in DM cases [3]. The global prevalence of TB in DM cases has increased 20-fold compared to that in non-DM cases, where 45% of DM sufferers reported experiencing TB complications [4,5].

The increased susceptibility of DM sufferers to TB disease is partly due to the high global prevalence of TB disease, especially in Indonesia, which is ranked third in the world [6]. DM susceptibility to TB infection is highly dependent on the ability of the DM individual’s immune response to eradicate TB, where the conditions of hyperglycemia and cellular insulinopenia contribute directly to impaired innate immune cell function, such as the decreased phagocytosis of macrophages, the impaired secretion of interleukin-12 (IL-12), the function of Toll-like receptor-2 (TLR-2), and the decreased expression of the molecule major histocompatibility complex class-II (MHC-II) [7], which causes DM sufferers to become easily infected with TB [8].

Previous studies have reported several problems with the innate immune response in DM, particularly macrophage cells. These cells act as the first line of defense against TB infection, such as the recognition process by TLR-2 and IL-12 cytokine secretion as an effector of calcic pathway macrophage activation, as well as MHC-II antigen presentation as a response. In terms of adaptive immunity, it is important to strengthen the function of macrophages in people with DM by administering immunomodulating compounds.

Administering immunomodulating compounds to people with DM is important to strengthen the innate immune response and ensure that they are not prone to complications with TB infection. It is also conducted to achieve TB eradication through the innate immune response mechanism by the macrophages, which is the first line of defense. The current lack of effective immunomodulating compounds is an obstacle to overcoming the problem of decreased immune system functioning in people with DM, so it is necessary to seek and develop immunomodulating compounds.

An alternative to the development of immunomodulatory compounds derived from natural materials, whose mechanism of action is known to modulate the innate immune system in DM and TB-infected DM people, is important. Several studies have shown that secondary metabolites isolated from plants, such as delphinidin, malvidin, resveratrol, and quercetin, have immunomodulatory activity [9,10].

The plant *Etlingera rubroloba* A.D. Poulsen was reported to contain secondary metabolites tannins, flavonoids, saponins, alkaloids, polyphenols, terpenoids, triterpenoids, and phenolics [11], as well as the isolated secondary metabolites from the stem extract of *E. rubroloba*, namely Sinaphyl alcohol diacetate [12,13]. Pharmacological activities of this plant have been reported, such as *E.rubroloba* stems, which act as antioxidants, xanthine oxidase inhibitors, and anti-inflammatories [12,13,14]. Extracts and fruit fractions of *E. rubroloba* are known as immunomodulators in DM mice infected with TB, increasing the phagocytic activity of macrophage cells and helper T lymphocytes (CD4) in vivo [11,15,16]. *E. rubroloba* fruit juice has been used by the Wawonii and Tolaki ethnic communities in southeast Sulawesi to increase immunity (immunomodulator) [17].

*E. rubroloba* is a newly discovered species and is endemic in southeast Sulawesi, so there are a few studies that report the content of secondary metabolites, isolated compounds, and their pharmacological activity, especially the immunomodulating effect on DM [18]. Therefore, in this research, we obtained natural compounds that are safe and effective immunomodulators and can be used for the development of new drugs, thereby providing a solution for DM sufferers to prevent complications with TB disease.

## 2. Results

### 2.1. Phagocytosis Activity and Levels of Interleukin-12 Extracts and Fractions In Vivo

Phagocytosis activity is determined by counting the number of macrophage cells that phagocytize antigens in 100 macrophage cells. The value of the phagocytosis of macrophages is expressed as a percentage, and IL-12 level examination is carried out using the sandwich ELISA method [16]. The measurement results are shown in Table 1.

Table 1 shows that the phagocytic activity level of macrophages was highest in the treatment group fraction C, followed by fractions A, D, B, E, and F, and it was the lowest in the negative control group (DM+TB). The statistical results of Tukey’s post hoc test showed no significant difference between the A, B, D, E, and F group fractions with the positive control (*p* > 0.05) in terms of macrophage phagocytosis activity and IL-12 levels. Meanwhile, the C fraction, ethyl acetate fraction, and extract showed no significant difference in macrophage phagocytosis activity and IL-12 levels with those of the positive control (*p* < 0.05), where the increased phagocytosis activity and decreased levels of IL-12 fraction C, ethyl acetate fraction, and the extract are better than those of the positive control.

### 2.2. Isolation and Purification of Fraction C

**Figure 1:**
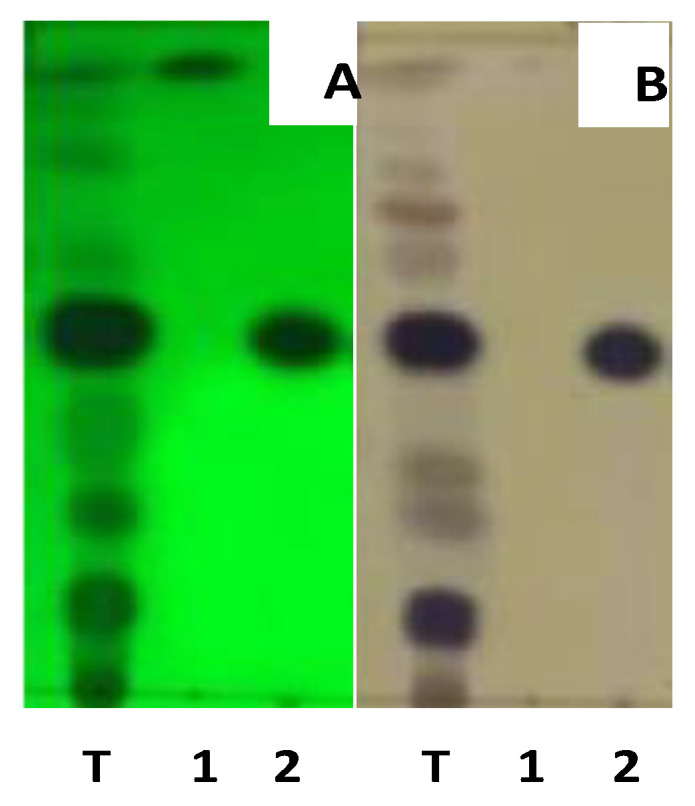
Chromatogram of purity test for isolates using three n-hexane solvent systems: ethyl acetate (7:3), (**A**) UV 254 nm; (**B**) cerium sulfate; fraction C (T); BER-1(1); BER-6 (2).

Fraction C from *E. rubroloba* fruit was the most active immunomodulator. Separation and purification of the secondary metabolites were carried out using TLC, VLC, and radial chromatography. Fraction C was separated for the first time by the VLC method using the eluent system optimized at TLC, namely n-hexane and ethyl acetate at ratios of (10:0); (9:1); (8:2); (7:3); (6:4); (5:5); (4:6); (3:7); (2:8); (1:9); (0:10) (*v*/*v*), showing a good separation pattern. There are two isolates that were obtained from the isolation of the secondary metabolites. The purity of the isolates was tested using three eluent systems: n-hexane: ethyl acetate (7:3), n-hexane: acetone (7:3), and n-hexane:chloroform: acetone (7:2:1), which showed that the chromatogram obtain by TLC showed a single spot. Thus, it was concluded that the isolated compound was pure, with the BER-1 weight being 37.7 mg and BER-6 weight being 32 mg. The results of the test can be seen in Figure 1.

### 2.3. Identification of Isolated Compound Structures

The two isolate compounds were successfully isolated from the secondary metabolites of the *E. rubroloba* fruit extract, then structural determination and identification were carried out based on 1D (^1^H, ^13^C) NMR spectroscopy data analysis. The results of the proton (^1^H) NMR spectra to determine the total number of protons include: the chemical shift (δH (ppm), multiplicity ((singlet (*s*), doublet (*d*), triplet (*t*), quartet (*q*), multiplet (*m*)), and the interpretation of the carbon (^13^C) NMR to determine the total amount of carbon in a compound. The interpretation of the results of the proton and carbon NMR of the isolated compounds can be seen in Table 2.

**Figure 2:**
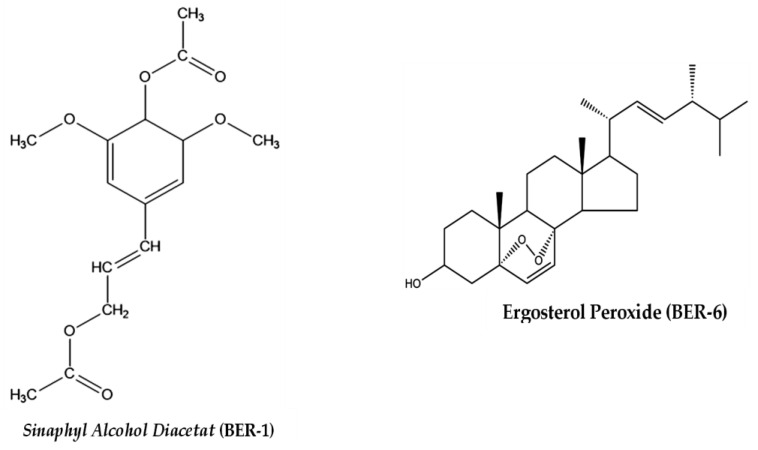
Structure of isolated and identified secondary metabolites from *E. rubroloba* fruit.

The interpretation of the ^1^H-NMR and ^13^C-NMR spectra of the isolate compounds in Table 2 and a comparison with the literature show that the BER-1 isolate is Sinaphyl alcohol diacetate, while BER-6 is the isolate, Ergosterol peroxide. The structural formulas of the compounds are shown in Figure 2.

### 2.4. Immunomodulating Effects of E. rubroloba Fruit In Vitro

The immunomodulation effect is based on the parameters of the IL-12 level, TLR-2 protein expression, and HLA-DR.

#### Effect of Extracts and Isolate Compounds on IL-12 Levels

**Figure 3:**
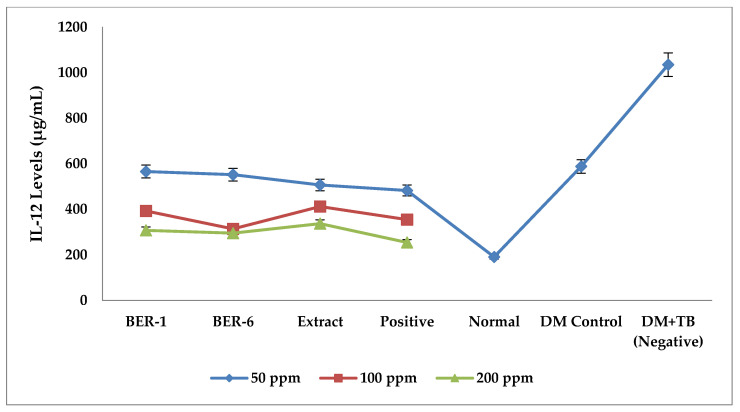
Graph of mean IL-12 levels of extracts and compounds isolated from *E.rubroloba* fruit at concentrations of 50, 100, and 200 (ppm) for each group.

The results of the measurement of IL-12 levels at various concentrations showed that the extracts and compounds isolated from *E. rubroloba* fruit exerted different immunomodulating effects based on the reduced levels of IL-12 (Figure 3).

The average IL-12 levels for each treatment in Figure 3 and Table 3 show that the highest IL-12 levels of all the groups were in the control group of DM and the DM+TB macrophage cells. The results of the Tukey LSD post hoc test can be seen in Table 3.

The results of Tukey’s post hoc test (Table 3) between the negative control group (DM+TB) with isolates and extracts were significantly different (* *p* < 0.05), indicating that there are immunomodulating potential of isolates and extracts, while the positive control with isolates and extracts showed significant differences in terms of the effect of reducing IL-12 levels (* *p* < 0.05).

### 2.5. Effects of Extracts and Isolate Compounds on TLR-2 Protein Expression

**Figure 4:**
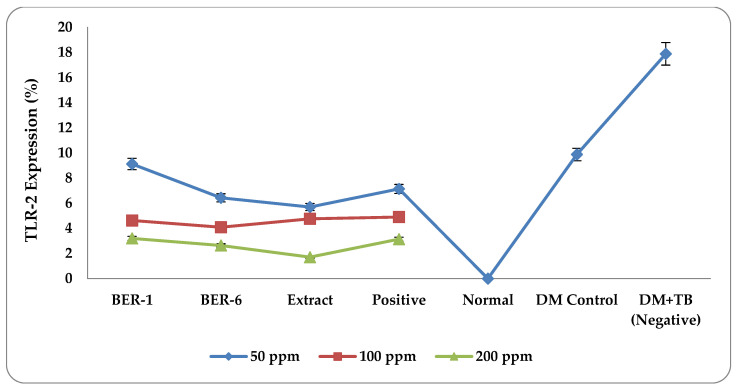
Graph of the mean results of measurements of TLR-2 expression of extracts and compounds isolated from *E. rubroloba* fruit at concentrations of 50, 100, and 200 (ppm) for each group.

The results of the measurements of TLR-2 protein expression with various concentrations, in Figure 3, show that the compounds and extracts from the fruit of *E. rubroloba* show different immunomodulation activities. The results of the measurements of the average TLR-2 protein expression in each treatment can be seen in Figure 4.

The graph of the average TLR-2 protein expression for each treatment in Figure 4 shows that of all the groups, the highest negative control value was for the DM+TB macrophage cell group. It also shows decreased TLR-2 expression in the isolate and extract groups. The post hoc test results (Table 4) of the negative control (DM+TB) and the extract and isolate groups show that there was a significant difference between them (* *p* < 0.05). This shows that the compound isolate and fruit extract of *E. rubroloba* have the potential to be immunomodulators by reducing the expression of the TLR-2 protein.

The results of the post hoc Tukey test were positive for the BER-1, BER-6, and extract groups. There were no significant differences (* *p* > 0.05) in the effect of reducing the TLR-2 expression; this explains that extracts and isolates from *E. rubroloba* fruit extract have the effect of reducing TLR-2 expression.

### 2.6. Effects of Extracts and Isolate Compounds on HLA-DR Protein Expression

**Figure 5:**
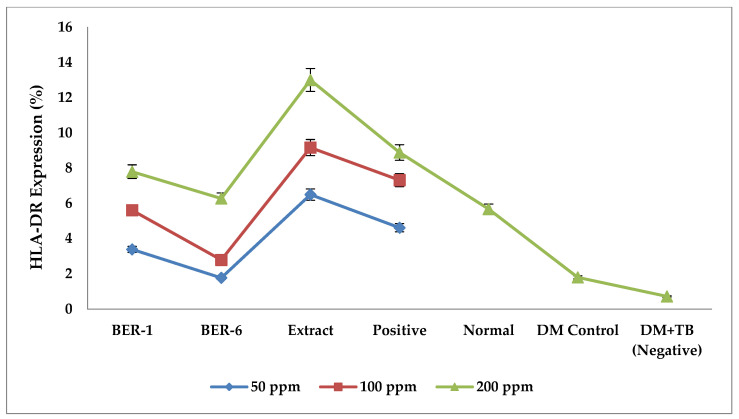
Graph of mean results of HLA-DR expression measurements of extracts and compounds isolated from *E. rubroloba* fruit at concentrations of 50, 100, and 200 (ppm) for each group.

The results of the HLA-DR expression measurements of various concentrations in Figure 5 show that the compound isolates and extracts from *E. rubroloba* fruit show different immunomodulation activities. The average HLA-DR protein expression for each treatment in Figure 5 and Table 5 show that the lowest HLA-DR protein expression values were in the negative control group (DM+TB) and DM control. The results of the measurement of the average HLA-DR expression in each treatment can be seen in Figure 5.

The highest mean HLA-DR protein expression was found in the extracts group, followed by BER-1, BER-6, and the positive control as compared to that of the negative control. The results of Tukey’s post hoc statistical test in Table 5 show that the isolates and extracts have a lot of potential as immunomodulators as compared to that of the negative control (* *p* < 0.05). The results of Tukey’s post hoc test for each treatment group can be seen in Table 5.

BER-1 isolates had the same effect of increasing the HLA-DR expression as the positive control did (* *p* > 0.05), while isolate BER-6 and the extract had the same effect of increasing the HLA-DR expression as the positive control did (* *p* < 0.05). The increase in HLA-DR expressions of the isolate and extract was larger than the positive control one was, so it can be concluded that the extract and isolates are effective as immunomodulators.

## 3. Discussion

Considering the increased phagocytic activity and the effect of decreasing IL-12 levels in DM model animals infected with TB, it is likely that there is a chemical compound contained in the extract and fruit fraction of *E. rubroloba*, which can improve the immune system function and anti-inflammatory effects in DM conditions. These results are supported by the research of Ilyas et al. [15], who reported that the ethanol extract and fraction of *E. rubroloba* fruit contains the compounds chavicol-β-D-glucoside, erigeside II, and 2-methoxyanofinic acid. These compounds are secondary metabolites of penylpropanoid-glycoside, terpenoid group, alkaloids, triterpenoids, and flavonoids, which have potential as immunomodulators and anti-inflammatories. These compounds have an immunostimulatory effect with mitogenic properties that stimulate the proliferation of T lymphocyte cells and B lymphocyte cells through the production of IL-12, IL-4, and IL-1 cytokines [15,19]. The proliferation of T lymphocyte cells will increase the activity of phagocytic cells such as macrophages [16].

We tested the immunomodulating effect of isolate compounds and extracts using in vitro methods on DM model macrophages infected with TB antigen using these parameters: IL-12 levels, TLR-2, and HLA-DR/MHC-II protein expression. The results showed that type 2 DM conditions showed an increase in IL-12, as an inflammatory condition that commonly occurs at the start of type 2 DM disease with long-term effects that can lead to complications such as tissue damage and a severe infection [20,21,22]. This study reaffirms previous research, which found that there was a larger increase in the IL-12 levels in the DM and DM-infected DM macrophage model groups (DM+TB) compared to those in the extract and isolate treatment groups. This is due to macrophage cells secreting more IL-12 at the start of TB infection to reduce the number of bacteria and control the development of TB disease [23].

Cytokine regulation in type 2 DM is very important, especially in the impact of complications of infectious diseases. It has been shown that the pro-inflammatory cytokine IL-12 has increased and is associated with acute inflammation at the start of type 2 DM [21]. Long-term increase in IL-12 has a negative impact on type 2 DM because it can cause organ or cell damage by inducing an oxidative stress-inflammation-dependent mechanism, which triggers oxidative stress and blood vessel damage by decreasing the expression of vascular endothelial growth factor receptor-2 [22].

The isolates and extracts in this study were shown to be able to regulate pro-inflammatory cytokines by reducing the IL-12 levels, which are needed to control excessive inflammatory responses in DM conditions and during TB infection and prevent more severe complications of TB infection [21]. During TB infection, a sufficient amount of IL-12 is needed to regulate the macrophages controlling TB pathogenicity in type 2 DM by secreting anti-bacterial cytokines, such as tumor necrosis factor (TNF), interferon-gamma (IFN-γ), and interleukin-6 (IL-6), as the effectors for cellular innate immune cells at overcoming an infection [24]. It can also increase the interaction of macrophage immunocompetent cells, NK cells, and T cells while fighting TB infection [25].

Inflammation has been shown to be a component of type 2 DM triggered through Toll-like receptor (TLR-2) signaling, in conjunction with free radical production, which is correlated with insulin resistance. Furthermore, TLR-2 and related signaling molecules in immune cells trigger increased inflammation in the adipose tissue of type 2 DM patients and contribute to the worsening of type 2 DM and susceptibility to complications with TB infection [26].

TLR-2 protein expression in this study showed larger increases in the DM and DM+TB model macrophage cell groups compared to those in the extract and compound isolate groups. TLR-2 expression in DM increased after the infection with TB antigens as a protective immune response by the host against TB infection. Furthermore, it initiated cellular innate immune responses to control the development of TB infection, which has been shown to have a negative impact such as complications in type 2 DM [27], as well as contribute to inflammatory events by signaling through DAMPs ligands [28,29]. Therefore, the regulation of TLR-2 in type 2 DM is important for controlling the inflammatory reaction that occurs. An increase in the amount of TLR-2 is directly related to the occurrence of an inflammatory response by increasing the pro-inflammatory cytokine IL-12, but on the other hand, the presence of a sufficient amount of TLR-2 is required by the host to protect against TB infection [26,30].

The research revealed that the expression of TLR-2 after the administration of isolates and extracts to DM model macrophage cells infected with TB antigens decreased compared to those of the DM and DM+TB groups. This study is in line with several previous studies that explained that a decrease in TLR-2 expression is needed to control excessive inflammatory responses in DM conditions or during TB infection and prevent more severe complications with TB infection [21,23]. A sufficient expression of TLR-2 protects the host during TB infection by regulating the pro-inflammatory and anti-inflammatory cytokines [31]. It plays a role in increasing the activity of phagocytic cells, which eliminate the TB infection, and modulate type 2 DM to prevent TB infection [32,33].

A sufficient amount of MHC/HLA-DR in type 2 DM regulates the immune response through the presentation of peptide epitopes from TB antigens, which are processed by the adaptive immune system to activate helper T lymphocyte cells to regulate the immune response to infection [34]. HLA-DR plays a role in NK cell activation through the expression of NKp30 and NKp46 receptors and causes the activation of adaptive immunity during TB infection [35]. The limited expression of HLA-DR affects the susceptibility of type 2 DM to complications related to the immune system, especially TB infection [36]. Type 2 DM shows a decrease in the expression of MHC-II/HLA-DR due to damage to the pancreas organ, causing a susceptibility to complications in type 2 DM [37] and contributing to the prognosis and the susceptibility to infectious complications [38].

This study reinforces the results of previous studies, where there was a larger increase in HLA-DR protein expression in DM+TB after were given isolates and extracts compared to those of the DM and DM+TB controls. The increased expression of HLA-DR in DM is very important to anticipate the risk of a susceptibility of type 2 DM to complications related to the immune system, such as TB infection [36]. It also plays a role in the activation of phagocytic cells, such as NK cells and macrophages, and causes the activation of adaptive immunity during TB infection [35]. Previous studies also reported that increasing the amount of HLA-DR had a protective effect against TB infection by increasing the activation of cytotoxic T lymphocytes (CD8) and helper T lymphocytes (CD4) [39,40].

The activity of the isolated compounds is thought to be influenced by the presence of functional groups in the molecular structure (Figure 2). Ketone groups, hydroxyl groups, and alkene groups in the aliphatic chain are found in the Sinaphyl alcohol diacetat (BER-1), which are derivatives of Sinaphyl alcohol from the phenylpropanoid group. The presence of ketone and hydroxyl groups in the aliphatic chain may have the effect of increasing the HLA-DR expression. The ketone and hydroxyl groups are not owned by the compound Ergosterol peroxide (BER-6), a steroid group compound that is thought to affect the increase in HLA-DR expression to a lesser extent than the BER-1 isolates do.

Based on the structure and activity relationship of the isolate compounds BER-1 and BER-6, the structural parts responsible for their pharmacological activity are the aromatic (diaryl) group, ketone, hydroxyl groups, and double bonds in the cyclic chain (heptane) [41]. It is this functional group that allows the pharmacological effect of the isolated compound to decrease IL-12 and TLR-2 protein expression and increase HLA-DR protein expression.

## 4. Materials and Methods

The research procedure involved several steps: sample collection and preparation, extraction and fractionation, screening tests for phagocytosis activity and levels of interleukin-12 (IL-12) extracts and fractions in vivo, the isolation and purification of isolate compounds, the identification of isolate compound structures, and tests of immunomodulation of extracts and isolates on in vitro DM macrophage models infected with TB antigens.

### 4.1. Sampling and Preparation of Samples

The samples used in this study were *E. rubroloba* fruit taken from Punggaluku Village, Laeya District, South Konawe Regency, southeast Sulawesi Province. The fruit sample (12.25 kg) was sorted while it was wet to separate out the foreign matter under running water. The sample was then dried to reduce the water content and prevent microbial growth and damage to the sample. Furthermore, simplicia chopping was carried out to increase the surface area of the sample, so that the extraction process of secondary metabolites in the sample during extraction process was maximized.

### 4.2. Extraction and Fractionation

A total 3.1 kg of *E. rubroloba* fruit simplicia powder was macerated with 96% ethanol for 3 × 24 h, with a solvent replacement every 24 h. The macerate was concentrated using a rotary vacuum evaporator at 50 °C and thickened in a water bath at 50 °C to obtain a viscous ethanol extract of 334.2 g, with a yield of 10.78%.

Fractionation of *E. rubroloba* fruit extract was carried out using vacuum liquid chromatography (VLC) method [42,43]. One hundred grams of extract was fractionated with VLC 4 times using n-hexane:ethyl acetate eluent obtained from eluent optimization. The results of the fractions from VLC were then tested by the thin-layer chromatography (TLC) method to determine the combination of fractions with the same Rf value and stain spots on the chromatogram. The resulting VLC fractions were then combined. Six main fractions were obtained and tested for TLC with n-hexane eluents: ethyl acetate (9:1), (7:3), and (6:4). Fractions A (1.18 g), B (3.18 g), C (4.01 g), D (3.86 g), E (23.94 g), and F (26.59 g) were then weighted. The obtained fractions were screened for immunomodulation activity in vivo with the parameters of macrophage cell phagocytosis activity and IL-12 levels to determine which fractions were effective as immunomodulators. Finally, isolation and purification of the most effective fraction were carried out.

### 4.3. Screening Test of Phagocytosis Activity and Levels of Interleukin-12 (IL-12) Extracts and Fractions In Vivo

Acclimatization and grouping of test animals: BALB/c male mice were adapted to the environment for 7 days in cages filled with husks. The number of mice was 5 per group, which was calculated based on the Federer formula [11].

Preparation of the test preparation: The extract and the fraction were suspended at a dose of 0.1 mg/kg bw in Na. 0.5% CMC, which was adjusted to the test animals’ body weight [15,16].

Preparation of Positive Control Preparations: The comparison of preparation used was 0.0005 g of commercial meniran (*Phyllanthus niruri* Linn.) extract in 30 mL of Na. CMC 0.5%, according to the dose given to mice. It was converted based on dose conversion calculations [44].

BALB/c Mice Models of Diabetes Mellitus: This research was approved by the Health Research Ethics Commission, Institute for Research and Community Service, Halu Oleo University (project number 706/UN29.20/PPM/2020). A total of 30 BALB/c male mice were modeled of DM by induced diabetogenic streptozotocin (Stz) dose of 150 mg/g bw intraperitoneally in Na citrate buffer, pH 6.0 [45]. The DM model of the test animals in this study was obtained 2 × 24 h after Stz induction with an average fasting glucose level of >200 mg/dL [11,46].

Treatment of BALB/c mice: A total of 40 DM and normal BALB/c mice were randomly divided into 10 groups. The test animals orally administered 0.5 mL/mice once day for 14 days [15,16] with the following treatment:

FA group: BALB/c mice were given fraction A at a dose of 0.1 mg/g bw;
FB group: BALB/c mice were given fraction B at a dose of 0.1 mg/g bw;
FC group: BALB/c mice were given fraction C at a dose of 0.1 mg/g bw;
FD group: BALB/c mice were given fraction D at a dose of 0.1 mg/g bw;
FE group: BALB/c mice were given fraction E at a dose of 0.1 mg/g bw;
FF group: BALB/c mice were given fraction F at a dose of 0.1 mg/g bw;
FEA group: BALB/c mice were given fraction Ethyl acetate at a dose of 0.1 mg/g bw;
BER-X group: BALB/c mice were given *E. rubroloba* fruit extract at a dose of 0.1 mg/g bw;
K+ group: BALB/c mice were given a commercial meniran (*Phyllanthus niruri* Linn.) extract suspension at a dose of 0.101 mg/g bw;
Group K-: BALB/c mice were given 0.5% Na-CMC suspension;
KN group: the control group of normal test animals was only given standard feed.

Macrophage Cell Phagocytosis Test: Each mouse was infected with 0.1 mL of BCG antigen intraperitoneally on the eighth day. After 3 h, the test animals were euthanized with 0.2 mL of ketamine HCL. Peritoneal fluid was taken, stained on a glass object, and fixed with methanol, then 10% giemsa stain was added. The preparations were observed under a microscope at a magnification of 10×–1000× [16].

Calculating Phagocytosis Activity of Macrophage Cells: The phagocytic activity of mouse peritoneal macrophage cells was calculated based on the value of phagocytosis activity (SPA), which is the percentage of macrophage cells that actively carry out the phagocytosis process in 100 macrophage cells [11,42].

(1) $$\mathrm{Phagocytic}\;\mathrm{Activity}=\frac{\mathrm{Amount}\;\mathrm{of}\;\mathrm{Active}\;\mathrm{Macrophage}\;\mathrm{Cells}}{\mathrm{Total}\;\mathrm{Macrophage}\;\mathrm{Cell}\;\mathrm{Amount}}\;\times \;100\;\%$$

### 4.4. Isolation and Purification of E. rubroloba Fruit Isolates

Separation and purification of secondary metabolites from *E. rubroloba* fruit were carried out in several stages.

Thin-Layer Chromatography (TLC): The concentrated extract was spotted on a TLC plate, then eluted with a mixture of n-hexane:ethyl acetate (9:1). The Rf value of each spot formed was calculated, and the separation was observed. The eluents with a well degree of separate of stains, used for the process of separate compounds [43,47].

Radial Chromatography (RC): The radial chromatography plate was inserted into the RC apparatus and moistened with eluent before use. The fruit fraction of *E. rubroloba* was injected into the plate using a syringe. The separation process of the compounds was observed using a 254–366 nm UV lamp.

Each separate component was collected in a vial, and its purity was determined using the TLC method in various eluent systems. Components with only a single stain after being tested by the TLC method were considered to be pure isolates [48].

### 4.5. Identification of Isolate Compound Structures

The pure isolate compound obtained from *E. rubroloba* fruit was determined by a spectroscopic technique. The spectrum was measured using an NMR spectrometer by measuring the ^1^H NMR and ^13^C NMR spectra determined using a JEOL JNM-ECZ500R/S1 FT NMR spectrometer (Japan) operating at 500.159 MHz (^1^H) and 125.765 MHz (^13^C) [43,49].

### 4.6. Immunomodulation Test of Extracts and Isolates in an In Vitro DM Model Stimulated with TB Antigen

Preparation of Extract and Test Isolates: The extracts and isolates were dissolved in 0.0025% *w/v* DMSO in sterile distilled water, then the extracts, isolates, and positive control compounds were prepared in a series of concentrations at 50, 100 and, 200 ppm [14,50]. One mL of each extract and compound isolate was prepared for testing the immunomodulation effect.

Preparation of RAW Macrophage Cell Line Culture of DM model: The RAW macrophage cell line culture resulting from 24 h incubation, 250 µL, was taken and transferred to a 75 cm^2^ container at 8 × 10^4^ cells/cm2 containing complete RPMI growth media. Then, anhydrous glucose (Gibco) concentration of 15 mMol was added to RPMI media containing 80% confluent growth macrophage RAW cells. Insulin levels in RAW macrophage cell cultures were measured to ensure that the DM model had been formed using an ELISA microplate reader. The DM model was formed when glucose levels were >120 mg/dL (7 mMol) or higher and the insulin levels decreased by 50% from the normal levels (normal insulin levels <12 mIU/L). DM model macrophage cells were then resuspended at a concentration of 250,000 cells/mL for further testing [51].

Expression Testing of TLR-2, HLA-DR with Flow Cytometry: Examination of TLR-2 and HLA-DR expression was carried out using the flow cytometry method with the TLR-2 recombinant rabbit monoclonal antibody anti-mouse kit (Invitrogen catalog no. MA5-32787) and the CD74 polyclonal antibody kit and fluorescein isothiocyanate (FITC) conjugate (BIOSS, catalog no. OS-2518R-FITC). The incubation media resulting from the RAW macrophage cell culture in the wells were added to each 400 µL of the test material in complete RPMI 1640 media, namely ethanol extract samples, isolate compounds, and positive controls at concentrations of 50, 100, and 200 (µg/mL), and a solvent control (DMSO 0.0025% w/v), in triplicate, then incubated in 5% CO_2_ incubator at 37 °C for 4 h. They were infected with ESAT-6 TB antigen at a concentration of 0.5 µg/mL (10^5^ CFU) [52], as much as 100 µL per well, then incubated in a 5% CO_2_ incubator at 37 °C for 60 min. By following the protocol in the kit, readings were taken using a FACS CaliburTM flow cytometer with a reading duration of 50,000 events. FACS data were read and analyzed based on the strategy of determining the boundaries and areas of macrophage cells with the FlowJoTM application. Furthermore, using a laser combination that captured the intensity of TLR-2, HLA-DR antibodies, macrophage populations with TLR-2, HLA-DR-positive characteristics were selected [53,54].

Testing IL-12 Levels with ELISA: The macrophage culture medium stimulated by Mtb antigen for all the treatment groups was transferred into an Effendorf tube, centrifuged at 1500 rpm for 5 min, and the supernatant was removed using a pipette for ELISA examination. IL-12 levels were examined using the ELISA method with the mouse IL-12 Elisa kit 96T (BT LAB catalog no. E2658Mo). The IL-12 examination procedure followed the protocol in the ELISA kit. Measurement of 96-well plate by ELISA reader at 450 nm wavelength [11].

### 4.7. Data Analysis

The data analysis method used in this study is one-way analysis of variance (ANOVA) method, with a 95% confidence level and a significance level (5% error rate (α = 0.05). Data analysis was performed with Tukey’s LSD post hoc analysis ANOVA and Tukey data analysis using the GraphPad Prism version 5.

## 5. Conclusions

This study found two active isolates of immunomodulating compounds in DM infected with TB, which significantly reduced the IL-12 levels and TLR-2 protein expression, where the average decrease in IL-12 levels and TLR-2 expression was lower than that of the positive controls. The HLA-DR protein expression showed a significant increase, which was higher than that of the positive control. These two isolates compounds were identified as Sinaphyl alcohol diacetat (BER-1), and Ergosterol peroxide (BER-6), which were recently reported to be found in *E. rubroloba* fruits, and are effective at immunomodulating DM infected with TB.

## Figures and Tables

**Table 1:** Screening results of macrophage cell phagocytosis activity and IL-12 levels in each group.

Groups	Phagocytic Activity Average ± SEM (%)	IL-12 Levels Average ± SEM (ng/mL)
FA (Fraction A 0.1 mg/g bw)	52.25 ± 2.30 *a	22.37 ± 0.18 *^a^
FB (Fraction B 0.1 mg/g bw)	50.55 ± 2.00 *a	23.11 ± 0.28 *a
FC (Fraction C 0.1 mg/g bw)	59.35 ± 1.71 *a,*b	19.09 ± 0.14 *a,*b
FD (Fraction D 0.1 mg/g bw)	51.0 ± 2.10 *a	21.02 ± 0.06 *a
FE (Fraction E 0.1 mg/g bw)	49.25 ± 1.81 *a	25.03 ± 0.12 *a
FF (Fraction F 0.1 mg/g bw)	47.00 ± 2.72 *a	28.05 ± 0.13 *a
FEA (Ethylacetate fraction 0.1 mg/g bw)	60.25 ± 2.00 *a,*b	21.22 ± 0.11 *a,*b
BER-X (Ethanol extract 0.1 mg/g bw)	63.75 ± 2.15 *a,*b	20.01 ± 0.11 *a,*b
K+ (Positive control 0.1 mg/g bw)	51.25 ± 2.16 *a	22.22 ± 0.65 *a
K- (Negative control/DM+TB)	29.67 ± 2.06	31.51 ± 0.40
Normal	0	15.467 ± 0.41

Description: *a: significantly different from the negative control (*p* < 0.05); *b: significantly different from the positive control (*p* < 0.05).

**Table 2:** The ^1^H and ^13^C NMR spectra of compounds BER-1 and BER-6.

No.C	BER-1			BER-6		
	δ _C_	δ _H_	(∑H, m, J (Hz))	δ _C_	δ _H_	(∑H, m, J (Hz))
1	134.73	-	-	37.0	-	-
2	103.30	6.63	(1H, *s*)	30.2	-	-
3	152.26	-	-	66.5	3.95	(1H, *m*)
4	128.63	-	-	51.2	-	-
5	152.26	-	-	79.4	-	-
6	103.30	6.63	(1H, *s*)	130.7	6.23	(1H, *d*, 8.0)
7	134.06	6.57	(1H, *d*, 15.85)	135.2	6.49	(1H, *d*, 8.0)
8	123.71	6.22	(1H, *dt*,15.87, 6.71)	82.1	-	-
9	64.97	4.70	(2H, *dd*, 6.10, 1.22)	51.7	-	-
10	170.97	-	-	34.7	-	-
11	21.12	2.32	(3H, *s*)	20.9	-	-
12	168.85	-	-	39.4	-	-
13	20.57	2.10	(3H, *s*)	44.6	-	-
14	56.21	3.82	(6H, *s*)	37.0	-	-
15	56.21	-	-	23.4	-	-
16	-	-	-	28.6	-	-
17	-	-	-	56.3	-	-
18	-	-	-	12.9	0.8	(3H, *s*)
19	-	-	-	18.2	0.87	(3H, *s*)
20	-	-	-	39.7	-	-
21	-	-	-	19.6	0.98	(3H, *d*, 7.0)
22	-	-	-	135.4	5.12	(1H, *dd*, 15.5, 8.5)
23	-	-	-	132.4	5.20	(1H, *ddd*, 7.5, 15.2)
24	-	-	-	42.8	-	-
25	-	-	-	23.1	-	-
26	-	-	-	20.6	0.82	(3H, *d*, 7.0)
27	-	-	-	19.9	0.81	(3H, *d*, 6.5)
28	-	-	-	17.5	0.89	(3H, *d*, 7.0)

**Table 3:** Mean levels of IL-12 ± SEM of extracts and isolates from *E.rubroloba* fruit in each treatment group.

Groups	IL-12 Levels Average ± SEM (µg/mL)
BER-1	422.32 ± 58 *a,*b
BER-6	454.04 ± 47 *a,*b
Extract	485.65 ± 36 *a,*b
Positive	272.12 ± 20 *a
Normal	190.91 ± 46.08 *a
DM control	763.33 ± 96.37 *a
DM+TB (negative)	1082.12 ± 178.17

Description: *a (significantly different from negative control); *b (significantly different from the positive control).

**Table 4:** Mean expression levels of TLR-2 ± SEM extracts and isolates from *E.rubroloba* fruit in each treatment group.

Groups	TLR-2 Expression Average ± SEM (%)
BER-1	5.31 ± 1.38 *a
BER-6	4.39 ± 0.41 *a
Extract	3.52 ± 0.72 *a
Positive	5.04 ± 0.85 *a
Normal	1.47 ± 0.49 *a
DM control	7.68 ± 0.42 *a
DM+TB (negative)	17.89 ± 1.10

Description: *a (significantly different from negative control); *b (significantly different from the positive control).

**Table 5:** Mean HLA-DR protein expression ± SEM in extracts and isolates from *E. rubroloba* fruit in each treatment group.

Groups	HLA-DR Expression Average ± SEM (%)
BER-1	5.6 ± 0.77 *a
BER-6	3.74 ± 0.65 *a,*b
Extract	9.57 ± 0.38 *a,*b
Positive	7.37 ± 0.73 *a
Normal	1.47 ± 0.49 *a
DM control	1.8 ± 0.34 *a
DM+TB (negative)	0.72 ± 0.29

Description: *a (significantly different from negative control); *b (significantly different from the positive control), significant.

## Data Availability

Not applicable.
